# Prevalence of Depression and Anxiety in Colorectal Cancer Patients: A Literature Review

**DOI:** 10.3390/ijerph16030411

**Published:** 2019-01-31

**Authors:** Yu-Ning Peng, Mei-Li Huang, Chia-Hung Kao

**Affiliations:** 1 Department of Medicine, College of Medicine, China Medical University, 404 Taichung, Taiwan; u102001403@cmu.edu.tw (Y.-N.P.); u103001309@cmu.edu.tw (M.-L.H.); 2 Graduate Institute of Biomedical Sciences and School of Medicine, College of Medicine, China Medical University, 404 Taichung, Taiwan; 3 Department of Nuclear Medicine and PET Center, China Medical University Hospital, 404 Taichung, Taiwan; 4 Department of Bioinformatics and Medical Engineering, Asia University, 413 Taichung, Taiwan

**Keywords:** colorectal cancer (CRC), depression, anxiety

## Abstract

*Background*: We aimed to review published studies to obtain the best estimate of the risk of depression and anxiety among colorectal cancer (CRC) patients. *Methods*: We searched the PubMed/Medline database, Web of Science, and Google Scholar on the prevalence of depression or anxiety in CRC patients. A review of 15 studies published between June 1967 and June 2018 were conducted, and 93,805 CRC patients were included. *Results*: The prevalence of depression among patients diagnosed with CRC ranged from 1.6%–57%, and those of anxiety ranged from 1.0%–47.2%. Studies in which an expert (psychiatrist) administered the interviews reported lower prevalence of both depression and anxiety. *Conclusion*: The findings of this review suggest that patients with CRC exhibited a significantly high prevalence of both depression and anxiety, and these symptoms can persist even after cancer treatment is completed. However, the correlation of age and the emergence of depression or anxiety in CRC patients still remain controversial.

## 1. Introduction

Colorectal cancer (CRC) is one of the most common cancers in men and women worldwide, representing approximately 10% of global cancer incidence [1]. According to the 2018 Global Cancer Statistics [2], CRC is the second leading cause of cancer-related mortality, with an estimated 881,000 cancer deaths worldwide in 2018. As the population rapidly ages, cancer incidence continues to rise. The highest incidence rates are found central and Northern Europe (Hungary, Slovenia, Slovakia, the Netherlands, and Norway), Australia, New Zealand, Northern America, and Eastern Asia (Japan, the Republic of Korea, and Singapore). Over 65% of new cases occur in developed countries, with almost half of all new cases estimated to occur in Europe and the Americas. Survival of CRC patients has significantly improved during the past decade due to earlier diagnosis and advances in chemotherapy, surgery, and radiation therapy.

However, as survival rates of CRC patients improve, new challenges emerge. Physical and mental health problems, usually secondary to the cancer and its treatment, are experienced by many cancer survivors [3,4]. Symptoms include depression, anxiety, fatigue, pain, and cognitive deficits [5,6,7,8]. Evidence suggests a contributory role of cancer and its treatment in these symptoms and in forms of psychological distress such as depression and anxiety [9].

Depression, anxiety, and bipolar disorders are common among patients diagnosed with cancer [8,10,11]. Cancer survivors can experience these symptoms for more than 10 years after treatment [12]. One review article revealed that depression and anxiety are highly associated with oropharyngeal, pancreatic, breast, and lung cancers, and are found in 13%–25% of CRC patients [13]. Another review indicated that the point prevalence of depression in CRC patients ranges from 13% to 57% [14] due to not only the low 5-year survival rate in CRC but also ileus, colostomy, and the adverse effects of chemotherapy, which overwhelm many patients.

Other studies have sought to address these problems. In this paper, we aimed to determine how common depression and anxiety are in people with CRC throughout the course of their treatment by conducting a review of the relevant published studies. We aimed to add more studies compared to previous reviews [13,14], and focused only on CRC patients. Moreover, we tried to determine the relevance of age to these types of psychological distress.

## 2. Material and Methods

### 2.1. Search Strategy

We searched for relevant studies evaluating the prevalence of depression and anxiety in CRC patients by using the National Library of Medicine’s PubMed database, Web of Science, and Google Scholar. MeSH terms and the keywords colon, rectal, colorectal, cancer, depression, depressive symptoms, mood disorder, psychological distress, anxiety, and mental illness were used in various combinations. The search was limited to studies published between June 1967 and June 2018. The reference lists of these studies were examined to identify additional studies. This review focused on studies with an adequate sample size and appropriate methodology. Only studies written in English and focusing on adult cancer survivors were included.

### 2.2. Inclusion Criteria

Two authors (Yu-Ning Peng and Mei-Li Huang) evaluated titles and abstracts of the studies searched initially, and the full articles were evaluated independently to determine whether they met our inclusion criteria. We limited our search to randomized controlled trials (RCTs) published in English and performed on humans. The included studies were selected based on the following inclusion criteria: (1) RCTs that aimed to estimate the prevalence of depression or anxiety in CRC patients; (2) all study participants were adults (>18 years old); (3) all study participants had a definite cancer diagnosis. Exclusion criteria were: studies that did not use standard diagnostic criteria [e.g., Minnesota Multiphasic Personality Inventory (MMPI), Hospital Anxiety and Depression Scale (HADS), International Classification of Diseases (ICD)…etc.] to evaluate depression or anxiety.

### 2.3. Data Analysis

We assembled studies identified in the initial search into a database and screened their titles and abstracts for relevance. We then reviewed the entire texts of potentially relevant articles. The following data were extracted from these studies: number and numbers of participants included, percentage of female participants, number of CRC patients, methods of depression and anxiety diagnosis, and prevalence of depression and anxiety among the participants.

## 3. Results

### 3.1. Study Selection

**Figure 1:**
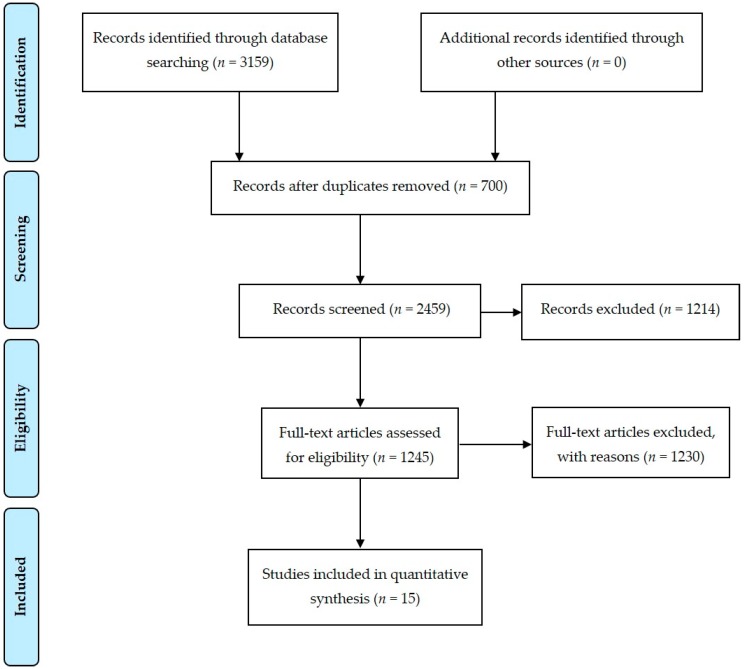
Article selection flow chart for the systematic review according to PRISMA (Preferred Reporting Items for Systematic Reviews and Meta-Analyses) guidelines.

The initial search and screening of titles yielded 2459 potentially eligible studies. After reviewing the abstracts of these studies, 2444 were deemed not relevant to the topic of this review. Fifteen studies published from 1967 to 2018 were thus eligible for inclusion (Figure 1). Their characteristics are listed in Table 1.

### 3.2. Characteristics of Study Participants

The included studies had been undertaken in ten different countries. Nine focused on patients with CRC; three included patients with gastrointestinal or digestive cancer; and the remaining three included patients with various cancer types.

### 3.3. Diagnosis of Depression and Anxiety

The diagnostic criteria for depression and anxiety varied among the included studies. The majority (eight studies) used the Hospital Anxiety and Depression Scale (HADS) for diagnosis, two studies used ICD-9, one study combined HADS and SCID-I, and the remaining studies used the Minnesota Multiphasic Personality Inventory (MMPI), Brief Symptom Inventory-18 (BSI-18), Veterans RAND 12 Item Health Survey (VR-12), or Beck Depression Inventory (BDI). Four studies employed a psychiatrist to conduct diagnostic interviews, whereas the other 11 used research assistants. Five studies assessed inpatients, six evaluated outpatients, and four included both inpatients and outpatients. The interviews were conducted either face-to-face or by telephone.

### 3.4. Prevalence of Depression and Anxiety in CRC Patients

The prevalence of depression and anxiety observed in each study is shown in Table 1 and Table 2. Fifteen studies from ten countries were included. The prevalence of depression among CRC patients ranged from 1.6% to 57%, and that of anxiety from 1.0% to 47.2%. Fras et al. published the first study on depression and anxiety [1]. This study included 110 US cancer patients, 64 of whom had received CRC diagnoses. The MMPI was used to screen patients for depression and anxiety, with results indicating that depression and anxiety were more frequent in patients with CRC than in the control group. The study indicated that the incidence of both depression and anxiety in CRC patients was 13%. The mean age of the patients was not presented in the study, but the authors mentioned that most patients were in their sixth and seventh decades (50–70 years old).

Of two studies by Japanese researchers from 2005, one evaluated depression in 85 inpatients with digestive cancers, of whom 38 had received CRC diagnoses and were awaiting surgery [19], and the other included 128 patients with CRC [20]. Both studies used HADS, a 14-item questionnaire (seven items each for the depression and anxiety subscales) designed for physically ill patients, to screen for depression. Each item is self-rated from 0–3, with a total subscale score ranging from 0 to 21. Matsushita et al. found that 28% of CRC patients had depression symptoms of at least moderate severity, but that only 8% of them were seeking treatment when the study was conducted [16]. The mean age of the patients was 68 ± 10.3 years (range, 44–87 years). Tsunoda et al. found that the prevalence of depression and anxiety in patients with CRC was 36.7% and 7.8%, respectively, and observed that depression had a stronger impact on overall quality of life than anxiety [15]. The mean age of the patients was 69 ± 10.5 years (range, 33–89 years), and 17% of the patients were below 60 years old, 40% were between 60 to 70 years old, and 43 % were over 70 years old.

Researchers in Australia [21] examined psychological distress among 1822 CRC patients through both telephone interviews and a self-administered questionnaire. The BSI-18 was used to assess psychological distress at 6 and 12 months post-diagnosis; the prevalence of depression was 7.5% at 6 months and 7.1% at 12 months, and that of anxiety was 7.4% at 6 months and 6.7% at 12 months. 28% of the CRC patients were <60 years old, 34% between 60–69 years old, and 38% over 70 years old. The authors suggested screening patients with CRC for distress at regular intervals to effectively detect those requiring in-depth psychological intervention. Tavoli et al. evaluated 142 patients with gastrointestinal cancer, 54 of whom had CRC [4]. All were being treated in a large teaching hospital (Imam Hospital) in Tehran, Iran. The mean age of the patients was 54.1 ± 14.8 years (range, 19–76 years). HADS was used in a face-to-face interview with a psychologist to measure depression and anxiety. Scores of 11 or higher were considered as indicating significant depression. The average time between cancer diagnosis and HADS evaluation was 4.4 months. The incidences of depression and anxiety were 57% and 47.2%, respectively, with no correlation to sex, education, marital status, or initial treatment (e.g., surgery and chemotherapy). The incidence of depression had no correlation with age; however, a significant relationship was observed between anxiety and age. Tavoli et al. demonstrated that young patients (<50 years) are more distressed than elderly patients (>50 years) when they have gastrointestinal cancer.

Zhang et al. investigated the ICD-9 diagnostic rates of depressive and anxiety disorders, including neurotic depression, adjustment disorder with depressed mood, major depression, and anxiety states, among 56,182 elderly US Medicare beneficiaries (age ≥ 65) who received a CRC diagnosis between 1998 and 2002 [3]. This was the largest number of patients of all the studies included in this review. The authors stated that ICD-9 diagnoses for depression and anxiety pertained to 1.6% and 1.0% of CRC outpatients, respectively. A total of 43 % of the patients were between the ages of 65 and 74 (defined as young-old in this study), 41% were 75 to 84 years old (mid-old), and 16.3% were over 85 years old (old-old). The author observed significantly higher rates of depression in elderly patients, especially in patients over 85 years old. The prevalence of anxiety was not correlated with age. In the same year, Medeiros et al. evaluated the prevalence of depression among 37 CRC patients after surgical resection, all of whom were being treated in the oncology division of Universidade Federal de São Paulo, Brazil [5]. The researchers used the BDI to assess depression in CRC patients, and found that its prevalence was 19.4%. The mean age of the patients in the chemotherapy group was 59 ± 11.5 years (range, 38–82 years), and in the control group, 65 ± 10.7 years (range, 38–82 years). They also observed that depression was more prevalent among patients receiving chemotherapy than in the control group. Hong et al. surveyed the prevalence of anxiety and depression among Chinese cancer patients [6]. They interviewed 1217 patients with various cancer types, including 103 patients with CRC. HADS was used to evaluate anxiety and depression status, and the resulting depression and anxiety prevalence rates were 54.4% and 2.9%, respectively. The mean age of the patients in the chemotherapy group was 51.2 ± 13.1 years. The authors concluded that depression was a more critical mental problem for cancer patients than anxiety.

Abu-Helalah et al. conducted a cross-sectional study of CRC survivors in Jordan who were diagnosed in 2009 and 2010 [7]. Of the 241 patients with CRC who completed the survey, the majority reported good to excellent overall health. Psychological distress among these patients was evaluated through a self-administered HADS questionnaire. The prevalence of depression and anxiety was 18% and 23%, respectively. The mean age of the patients was 56.7 ± 13.6 years old. In the same year, researchers from Scotland assessed the prevalence of depression in 21,151 patients with cancer in various primary sites, among whom 3355 had CRC. The prevalence of depression was highest in patients with lung cancer (13.1%, 95% confidence interval (CI): 11.9–14.2%), whereas among patients with CRC, it was 7.0% (95% CI: 6.1–8.0). The mean age of the patients was 64.4 ± 11.9 years (range, 19–100 years). In total, 31% of the patients were <60 years old, 32% were between 60–69 years old, and 36% were >70 years old. The authors also noted that 1130 (73%) of the 1538 patients with depression were not receiving effective treatment. Nikbakhsh et al. evaluated the prevalence of depression and anxiety among 150 patients in Iran with recent diagnoses of different cancer types, including 24 with CRC [8]. The HADS results indicated that the prevalence of depression was 16.9 % (7.5% mild and 9.4% symptomatic depression), whereas that of anxiety was 15.4 % (11.4% mild and 4.0% symptomatic anxiety). The mean age of the patients was 59 ± 14.3 years (range, 22–88 years). There were significant relationships between anxiety, depression and the age group of the patients (*p* = 0.004 and 0.007, respectively). They concluded that older age may increase the duration of disease and higher probability of cancer metastasis. These conditions might increase prevalence of anxiety and depression. Among all patients with cancers in different primary sites (breast, colorectal, stomach, esophagus, lung, and thyroid), patients with breast and stomach cancer had the highest prevalence of anxiety and depression.

In 2015, a study was conducted in Turkey including 105 patients with CRC by using HADS to assess depression and anxiety [18]. Life quality and sexual satisfaction were also evaluated using the European Organization for Research on Treatment of Cancer Quality of Life Questionnaire C30 and the Golombok–Rust Inventory of Sexual Satisfaction. The prevalence of depression and anxiety was 44% and 29%, respectively. The mean age of the patients was 52.9 ± 9.0 years (range, 28–76 years). The authors also demonstrated a significant association of depression and anxiety symptoms with quality of life scores and sexual dysfunction. Sexual dysfunction was considerably more common in patients with high depression and anxiety scores.

Clark et al. conducted a study including 1785 resected CRC patients by using VR-12 to evaluate prevalence of depression [9]. The mean age of the patients was 78 ± 7 years. The prevalence of depression was 15.6 %. The authors performed univariate analysis to identify correlates of positive depression screen in resected CRC survivors, and found that patients reporting male sex, higher education, home ownership, and were married were less likely to be associated with positive depression screen (all *p* < 0.05). However, age was not a factor associated with depression (*p* = 0.237).

Sun et al. performed a population- based cohort study using the Taiwanese National Health Insurance Research Database [10]. The study included 27,242 patients diagnosed with CRC between January 1, 2000, and December 31, 2010. Patients with CRC were found to have a significantly higher risk of developing mood disorders (adjusted hazard ratio (HR) = 3.05, 95% CI: = 2.89–3.20) compared with control patients. Among mood disorders, the prevalence of depression and anxiety was 6.6% and 32.5%, respectively. The mean age of the patients was 64.2 ± 13.5 years (range, 22–88 years). In total, 49% of the patients were below 65 years old, and 51% were over 65 years old. The authors also observed that patients with CRC were at particularly higher risk of mood disorders when the follow-up time was longer than 1 year. However, this association was affected by the type of treatment.

Finally, a population-based longitudinal study was conducted in the United Kingdom including 2625 patients with CRC diagnosed between 2000 and 2009 [11], which was the most recent study available. Using HADS for measurement, a higher prevalence of depression (19.0% vs. 12.8%) and anxiety (20.9% vs. 11.8%) was reported among patients with CRC compared with the control group. The mean age of the patients was 69.4 ± 9.5 years. The authors noted that elderly patients were associated with more depression, but interestingly with less anxiety. The authors also noted that symptoms of depression and anxiety were especially common among patients who were single, had a lower education level, and had comorbidities.

### 3.5. Relationship between Age and the Prevalence of Depression and Anxiety

Three studies [3,8,11] concluded that older patients were associated with higher levels of depression. Two studies [4,12] concluded that young age was associated with higher prevalence of depression. Only one study [8] identified relationships of anxiety with age, and concluded higher prevalence of anxiety in elderly patients. One study [9] pointed out that age was not associated with depression, and one study [3] also found that age was not correlated with anxiety. The remaining studies did not mention the role of age in the emergence of depression and anxiety.

### 3.6. Quality Assessment

Two authors (Peng and Huang) independently evaluated each trial according to the JBI (Joanna Briggs Institute) critical appraisal checklist for studies reporting prevalence data; the methodological quality across all included trials studies ranged from 5 to 9, with higher score indicating lower risk of bias. Discrepancies in scoring were discussed among the authors. Nine studies (60%) scored 9 points, and one study socred 5 points. Quality assessment of the included trials is summarized in Table 3.

## 4. Discussion

The main finding of our review was that CRC patients had a significantly high prevalence of both depression and anxiety. We reviewed 15 studies on the prevalence of depression in CRC patients, 11 of which also examined the prevalence of anxiety. These studies reported a prevalence of depression ranging from 1.6% to 57% and a prevalence of anxiety ranging from 1.0% to 47.2%. The significance of age in the emergence of depression or anxiety in CRC patients still remains controversial. Most included studies did not report relationships between depression, anxiety and age. Moreover, we noted that studies in which a psychiatrist administered the interviews [3,10,12] reported a lower prevalence of both depression and anxiety than did those that employed research assistants, despite the fact that all the included studies used standard diagnostic criteria to assess psychological illness. The highest rates of depression and anxiety measured were 57% and 47.2 %, respectively, both from the same study [22], which evaluated 54 inpatients with CRC in Iran. Similarly, the lowest rates of depression and anxiety, measured at 1.6% and 1.0%, respectively, were both reported in the same study [23], which surveyed 56,182 elderly US Medicare beneficiaries with a CRC diagnosis and was also the largest study included in this review.

To the best of our knowledge, this is the first review examining the prevalence of both depression and anxiety in patients with CRC. Several reviews have focused on the prevalence of depression in patients with cancer. Massie et al. reviewed 88 studies and found that depression was highly associated with oropharyngeal, pancreatic, breast, and lung cancers, ranging from 1.5% to 57 %, with a lower prevalence in patients with colon and gynecological cancers or lymphoma (13%–25%) [13]. Many studies have sought to evaluate depression in cancer patients, but estimates of its prevalence vary widely. Walker et al. identified 66 relevant studies, of which only 15 met the quality criteria [12]. These studies estimated that the prevalence of depression was 5% to 16% in outpatients, 4% to 14% in inpatients, 4% to 11% among mixed outpatients and inpatients, and 7% to 49% in palliative care. Depression and anxiety in cancer patients substantially affect health functions and mortality risk. Among several meta-analyses that have reported on this, one comprising 25 observational studies indicated that mortality rates were as much as 25% higher in patients with cancer who had depressive symptoms. Chida et al. analyzed 165 relevant studies and found that depression was associated with higher mortality in both community-based cancer survivors (relative risk (RR) = 1.34) and in patients with cancer (RR = 1.08) [24]. Another meta-analysis [19] reported evidence that depression predicts mortality, but not progression, in cancer patients. Estimates were a greater mortality rate of 26% among patients with depressive symptoms and a 39% higher mortality rate among patients diagnosed with major depression. Among studies focusing on the prevalence of depression in patients with CRC, Sehlo et al., reviewed 6 studies and found that prevalence ranged from 13% to 57% [20]. The authors also noted that from one-quarter to one-third of all patients with CRC in Saudi Arabia experienced depressive disorders.

Mood symptoms are important issues for clinicians who care for CRC patients. Psychological distress is associated with mortality, morbidity and quality of life in cancer patients [21,22], and most of them are older people. A large prospective population-based study on elderly CRC patients examined the association between depressive symptoms and mortality among cancer survivors up to 10 years post-diagnosis [11]. They sub-analyzed CRC survivors and showed that depressive symptoms among 1- to 10-year CRC survivors and 1- to 2-year CRC survivors increased the mortality rate. Surprisingly, the suicide risk in elderly CRC patients is low (<0.2%), and no difference was found based on location of the primary tumor [23].

There are some limitations to this review. First, diagnostic methods for depression and anxiety varied among the included studies. Second, although some studies employed a psychiatrist or psychologist to conduct the diagnostic interviews, most studies employed research assistants. This may have resulted in deviations in the interview results. Third, a more structured approach to search for included trials must be conducted in order to find higher quality studies, and avoid random variables between studies.

## 5. Conclusions

In conclusion, depression and anxiety are common in patients with CRC, and can endure even after cancer treatment is completed. Depression and anxiety are challenging to study because the symptoms of psychological illness often overlap with fatigue or physical pain, and are difficult to differentiate. Symptoms of depression range from sadness to major depressive disorders. Consequently, a variety of screening tools are available to assess depression, anxiety, and other psychological illnesses. Depression and anxiety not only result in significantly higher mortality among patients with cancer, but also impair their quality of life during cancer treatment. Future studies should focus on establishing standard instruments for measuring depression and anxiety, and further investigating the prevalence of depression and anxiety in other diseases.

## Figures and Tables

**Table 1:** Characteristics of included studies for prevalence of depression.

Included Studies	Participants	*N*	Female (%)	CRC Patients	Mean Age (Years)	Methods	Investigators	In/Out Patients	Prevalence of Depression (%)
Fras et al., 1967, USA [1]	Gastrointestinal cancer	110	Not cited	64	N/A	MMPI	Research assistants	Inpatients	13
Tsunoda et al., 2005, Japan [15]	Colorectal cancer	128	42.0	128	69 ± 10.5	HADS	Research assistants	Outpatients	36.7
Matsushita et al., 2005, Japan [16]	Digestive cancer	85	37.6	38	68.0 ± 10.3	HADS	Research assistants	Inpatients	28
Lynch et al., 2008, Australia [17]	Colorectal cancer	1822	40.0	1822	Only age stratification	BSI-18	Research assistants	Outpatients	7.5 (6 months) 7.1 (12 months)
Tavoli et al., 2007, Iran [4]	Gastrointestinal cancer	142	44.4	54	54.1 ± 14.8	HADS	Psychologist	Inpatients	57
Zhang et al., 2010, USA [3]	Colorectal cancer	56,182	53.4	56,182	Only age stratification	ICD-9	Psychiatrist	Outpatients	1.6
Medeiros et al., 2010, Brazil [5]	Colorectal cancer	37	56.8	37	59 ± 11.5 (chemotherapy group) 65 ± 10.7 (control)	BDI	Research assistants	Inpatients	19.4
Hong et al., 2014, China [6]	Mixed	1217	40.3	103	51.2 ± 13.1	HADS	Research assistants	Inpatients	54.4
Abu-Helalah et al., 2014, Jordan [7]	Colorectal cancer	241	47.7	241	56.7 ± 13.6	HADS	Research assistants	Outpatients	18
Walker et al., 2014, UK [12]	Mixed	21,151	71.0	3355	64.4 ± 11.9	HADS/SCID-I	Psychiatrist	Inpatients/Outpatients	7
Nikbakhsh et al., 2014, Iran [8]	Mixed	150	52.0	24	59.0 ± 14.3	HADS	Research assistants	Inpatients/Outpatients	16.9
Akyol et al., 2015, Turkey [18]	Colorectal cancer	105	31.0	105	52.9 ± 9.0	HADS	Research assistants	Outpatients	44
Clark et al., 2016, USA [9]	Resected colorectal cancer	1785	50.8	1785	78.0 ± 7.0	VR-12	Research assistants	Inpatients/Outpatients	15.6
Sun et al., 2017, Taiwan [10]	Colorectal cancer	135,288	39.0	27,242	64.2 ± 13.5	ICD-9	Psychiatrist	Inpatients/Outpatients	6.6
Mols et al., 2018, UK [11]	Colorectal cancer	2625	44.9	2625	69.4 ± 9.5	HADS	Research assistants	Outpatients	19

MMPI = Minnesota Multiphasic Personality Inventory; HADS = Hospital Anxiety and Depression Scale; BSI-18 = Brief Symptom Inventory-18; ICD-9 = International Statistical Classification of Diseases and Related Health Problems, 9th Revision; BDI = Beck Depression Inventory; SCID-I = Structured Clinical Interview for DSM-IV Axis I Disorders; VR-12 = Veterans RAND 12-Item Health Survey.

**Table 2:** Characteristics and analyses of included studies for prevalence of anxiety.

Included Studies	Participants	*N*	Female (%)	CRC Patients	Mean Age	Methods	Investigators	In/Out-Patients	Prevalence of Anxiety (%)
Fras et al., 1967, USA [1]	Gastrointestinal cancer	110	Not cited	64	N/A	MMPI	Research assistants	Inpatients	13
Tsunoda et al., 2005, Japan [15]	Colorectal cancer	128	42.0	128	69 ± 10.5	HADS	Research assistants	Outpatients	7.8
Lynch et al., 2008, Australia [17]	Colorectal cancer	1822	40.0	1822	Only age stratification	BSI-18	Research assistants	Outpatients	7.4 (6 months) 6.7 (12 months)
Tavoli et al., 2007, Iran [4]	Gastrointestinal cancer	142	44.4	54	54.1 ± 14.8	HADS	Psychologist	Inpatients	47.2
Zhang et al., 2010, USA [3]	Colorectal cancer	56,182	53.4	56,182	Only age stratification	ICD-9	Psychiatrist	Outpatients	1.0
Hong et al., 2014, China [6]	Mixed	1217	40.26	103	51.2 ± 13.1	HADS	Research assistants	Inpatients	2.9
Abu-Helalah et al., 2014, Jordan [7]	Colorectal cancer	241	47.7	241	56.7 ± 13.6	HADS	Research assistants	Outpatients	23
Nikbakhsh et al., 2014, Iran [8]	Mixed	150	52.0	24	59.0 ± 14.3	HADS	Research assistants	Inpatients/Outpatients	15.4
Akyol et al., 2015, Turkey [18]	Colorectal cancer	105	31.0	105	52.9 ± 9.0	HADS	Research assistants	Outpatients	29
Sun et al., 2017, Taiwan [10]	Colorectal cancer	135,288	39.0	27,242	64.2 ± 13.5	ICD-9	Psychiatrist	Inpatients/Outpatients	32.5
Mols et al., 2018, UK [11]	Colorectal cancer	2625	44.9	2625	69.4 ± 9.5	HADS	Research assistants	Outpatients	20.9

MMPI = Minnesota Multiphasic Personality Inventory; HADS = Hospital Anxiety and Depression Scale; BSI-18 = Brief Symptom Inventory-18; ICD-9 = International Statistical Classification of Diseases and Related Health Problems, 9th Revision.

**Table 3:** Quality assessment of the included trials following the JBI (Joanna Briggs Institute) Critical Appraisal Tool.

Included Studies	Item 1	Item 2	Item 3	Item 4	Item 5	Item 6	Item 7	Item 8	Item 9	Score
Fras et al., (1967) [1]	Unclear	No	Yes	No	Yes	Yes	Unclear	Yes	Yes	5/9
Tsunoda et al., (2005) [15]	Yes	Yes	Yes	Yes	Yes	Yes	Yes	Yes	Yes	9/9
Matsushita et al., (2005) [16]	Unclear	Yes	Yes	Yes	Yes	Yes	Yes	Yes	Yes	8/9
Lynch et al., (Australia) [17]	Yes	Yes	Yes	Unclear	Yes	Yes	Yes	Yes	Yes	8/9
Tavoli et al., (2007) [4]	Unclear	Unclear	Yes	Yes	Yes	Yes	Yes	Yes	Yes	7/9
Zhang et al., (2010) [3]	Yes	Yes	Yes	Unclear	Yes	Yes	Yes	Yes	Yes	8/9
Medeiros et al., (2010) [5]	Yes	Yes	No	Yes	Yes	Yes	Yes	Yes	Yes	8/9
Hong et al., (2014) [6]	Yes	Yes	Yes	Yes	Yes	Yes	Yes	Yes	Yes	9/9
Abu-Helalah et al., (2014) [7]	Yes	Yes	Yes	Yes	Yes	Yes	Yes	Yes	Yes	9/9
Walker et al., (2014) [12]	Yes	Yes	Yes	Yes	Yes	Yes	Yes	Yes	Yes	9/9
Nikbakhsh et al., (2014) [8]	Yes	Yes	Yes	Yes	Yes	Yes	Yes	Yes	Yes	9/9
Akyol et al., (2015) [18]	Yes	Yes	Yes	Yes	Yes	Yes	Yes	Yes	Yes	9/9
Clark et al., (2016) [9]	Yes	Yes	Yes	Yes	Yes	Yes	Yes	Yes	Yes	9/9
Sun et al., (2017) [10]	Yes	Yes	Yes	Yes	Yes	Yes	Yes	Yes	Yes	9/9
Mols et al., (2018) [11]	Yes	Yes	Yes	Yes	Yes	Yes	Yes	Yes	Yes	9/9

Item 1 = appropriate sample frame to address the target population; Item 2 = appropriate way to sample study participants; Item 3 = adequate sample size; Item 4 = study subjects and the setting described in detail; Item 5 = data analysis conducted with sufficient coverage of the identified sample; Item 7 = valid methods used for the identification of the condition; Item 7 = measured in a standard, reliable way for all participants; Item 8 = appropriate statistical analysis; Item 9 = appropriate response rate.
